# Improvement of dose calculation in radiation therapy due to metal artifact correction using the augmented likelihood image reconstruction

**DOI:** 10.1002/acm2.12325

**Published:** 2018-04-17

**Authors:** Christian Ziemann, Maik Stille, Florian Cremers, Thorsten M. Buzug, Dirk Rades

**Affiliations:** ^1^ Department of Radiotherapy University Medical Center Schleswig Holstein/Campus Luebeck Luebeck Germany; ^2^ Institute of Medical Engineering University of Luebeck Luebeck Germany

**Keywords:** dose calculation accuracy, metal artifact reduction, radiotherapy

## Abstract

**Background:**

Metal artifacts caused by high‐density implants lead to incorrectly reconstructed Hounsfield units in computed tomography images. This can result in a loss of accuracy in dose calculation in radiation therapy. This study investigates the potential of the metal artifact reduction algorithms, Augmented Likelihood Image Reconstruction and linear interpolation, in improving dose calculation in the presence of metal artifacts.

**Materials and Methods:**

In order to simulate a pelvis with a double‐sided total endoprosthesis, a polymethylmethacrylate phantom was equipped with two steel bars. Artifacts were reduced by applying the Augmented Likelihood Image Reconstruction, a linear interpolation, and a manual correction approach. Using the treatment planning system Eclipse™, identical planning target volumes for an idealized prostate as well as structures for bladder and rectum were defined in corrected and noncorrected images. Volumetric modulated arc therapy plans have been created with double arc rotations with and without avoidance sectors that mask out the prosthesis. The irradiation plans were analyzed for variations in the dose distribution and their homogeneity. Dosimetric measurements were performed using isocentric positioned ionization chambers.

**Results:**

Irradiation plans based on images containing artifacts lead to a dose error in the isocenter of up to 8.4%. Corrections with the Augmented Likelihood Image Reconstruction reduce this dose error to 2.7%, corrections with linear interpolation to 3.2%, and manual artifact correction to 4.1%. When applying artifact correction, the dose homogeneity was slightly improved for all investigated methods. Furthermore, the calculated mean doses are higher for rectum and bladder if avoidance sectors are applied.

**Conclusion:**

Streaking artifacts cause an imprecise dose calculation within irradiation plans. Using a metal artifact correction algorithm, the planning accuracy can be significantly improved. Best results were accomplished using the Augmented Likelihood Image Reconstruction algorithm.

## 1 INTRODUCTION

In radiotherapy, computed tomography (CT) images are used to calculate dose distributions within a heterogeneous tissue. A basic requirement for accurate planning is the correct reconstruction of Hounsfield units (HU) and the corresponding electron or mass density.^1^, ^2^ Beam hardening, noise, scatter, and photon starvation caused by metallic objects within the patient body cause artifacts, which result in inconsistencies within the CT raw data. The majority of CTs use filtered back projection (FBP) for image reconstruction, which assumes consisting raw data. During the reconstruction, the method smears back the inconsistent projection values into the image by utilizing the back projection operation. This results in streaking artifacts reducing the image quality.^3^, ^4^, ^5^ Anatomical details may be superimposed by metal artifacts and are therefore not distinguishable from each other. Furthermore, values for electron density might be too high or too low in some regions due to the occurrence of artifacts. Consequently, these distorted values are directly influencing the calculation of dose distribution.

Several studies show that applying a metal artifact reduction (MAR) algorithm can minimize errors in dose calculation. In 2015, Baer et al. reported a dose difference of up to ±5% in the target volume and organs at risk for a head and neck patient with dental fillings when comparing patient plans with corrected and uncorrected images.^6^ In 2013, Spadae et al. investigated the ability to restore correct HU values by a MAR algorithm in the presence of titanium and cerrobend within a phantom. The study showed that the error made in a Monte Carlo calculation based on corrected and uncorrected image data strongly depends on the mass number of the material used in the implant. In this way, they showed that the error in dose calculation was up to 23.56% for the planning target volume (PTV) region in the case of cerrobend. However, the error could be reduced to 0.11% by a MAR algorithm. In conclusion, it is stated that the dose calculation is more accurate when the reconstructed density information is less distorted.^7^ In this context, the ability to retrieve correct HU values should be the main evaluation criterion for a MAR algorithm.^6^, ^7^, ^8^, ^9^, ^10^, ^11^

In a previous study, we investigated the MAR algorithm Augmented Likelihood Image Reconstruction (ALIR) regarding its ability to retrieve attenuation coefficients in the presence of streaking artifacts.^12^ It was shown that ALIR is able to correct distorted HU values to a high accuracy. Furthermore, structures that were not perceptible due to streaking artifacts were reconstructed accurately using ALIR.

In the current study, we investigate the impact of corrected HU values on the dose calculation after applying different MAR algorithms. A PMMA phantom that imitates a patient with double‐sided total endoprosthesis (TEP) was created. Two different case scenarios were investigated. In the first scenario, we calculate volumetric modulated arc therapy (VMAT) plans with double arc rotation, without taking into account possible influence of the steel inserts on the dose distribution during the planning process. In the second scenario, dose effects near the implant and the shadowing characteristic of the steel inserts are minimized using avoidance sectors, which avoid direct irradiation through the implants.^13^ In order to investigate the dosimetric effects, plans based on metal artifact corrected and original reconstructed images are compared. Furthermore, a measurement of the applied dosage within the isocenter of the phantom based on ionization chambers is used as a reference.

## 2 MATERIALS AND METHODS

### 2.A Phantom

**Figure 1:**
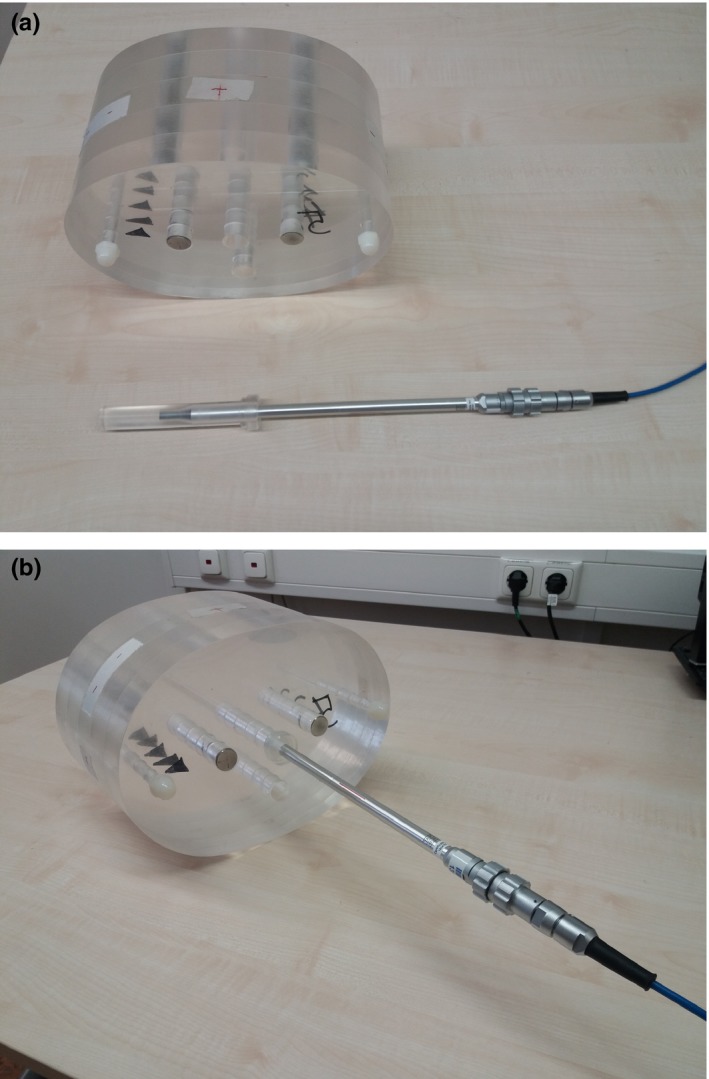
(a) PMMA phantom and ionization chamber. (b) PMMA phantom with mounted ionization chamber.

In order to simulate a pelvis with a double‐sided endoprosthesis for the hip, a PMMA phantom (Fig. 1) was manufactured that features two steel rods (CrNiMo, diameter Ø = 2.0 cm, length l = 15.0 cm).

The phantom consists of five layers (height 3.0 cm) with an elliptical base (half‐axes a = 20.0 cm, b = 28.0 cm). The stacked layers are held together by two laterally arranged rods of PMMA (Ø = 1.0 cm, l = 18.0 cm) that pass through all plates. Another bore with a diameter of 2.0 cm is used to simulate a rectum filled with air. Furthermore, the phantom can be equipped with an ionization chamber as shown in Figs. 1(a) and 1(b).

### 2.B CT imaging

For the acquisition of images, a 40‐slice CT scanner type Biograph mCT (Siemens AG, Erlangen, Germany) was used. The scans were acquired sequentially with 120 kVp, a field of view of 500 mm, and a slice thickness of 4 mm. The images were reconstructed using the filtered backprojection with a ramp filter (FBP) and the iterative algorithm ALIR (see next paragraph).

In addition to a dataset with the two metal rods, an image dataset without steel rods was acquired in order to have an artifact‐free image set available. This was used for the contouring of the ionization chamber.

The Hounsfield scale in clinical use is usually limited to a maximal value of 3071 HU. This is sufficient to represent the organs and bones according to their specific densities. However, in order to cover the steel inserts, the used Hounsfield scale was extended to 13,500 HU. This corresponds to a material density of *ρ*_(Steel)_ = 7.9 g/cm³.

### 2.C Artifact correction

The reduction in metal artifacts is performed utilizing three different approaches. The linear interpolation approach (LI) represents a simple and easy applicable reference method for the reduction in artifacts. Here, for every angle, projections that pass through the metal object are replaced by a linear interpolation between uncorrupted projection values.^14^ Based on the resulting raw data, the reconstruction of the image is performed using the FBP.

As a second approach, the recently proposed ALIR algorithm is used.^15^ The method is based on an iterative scheme and integrates two different ideas in order to reduce streaking artifacts. In a first step, the algorithm formulates the reconstruction of an image as an optimization problem based on the negative log‐likelihood function for transmission CT.^16^, ^17^ The optimization process is complemented by constraints that force the reconstruction to assign certain attenuation values in the region of the metal implant. In the present case, values are derived from the used steel rods. Furthermore, the ALIR algorithm exploits the iterative character of the optimization process to calculate new projection values that are used instead of the originally measured values. In each iteration of the algorithm, the interim result is filtered by a bilateral image filter to reduce occurring artifacts. The resulting image is used to calculate new projection values that are associated with the metal object. These values are then used in combination with the originally uncorrupted projection values for the next iteration.

**Figure 2:**
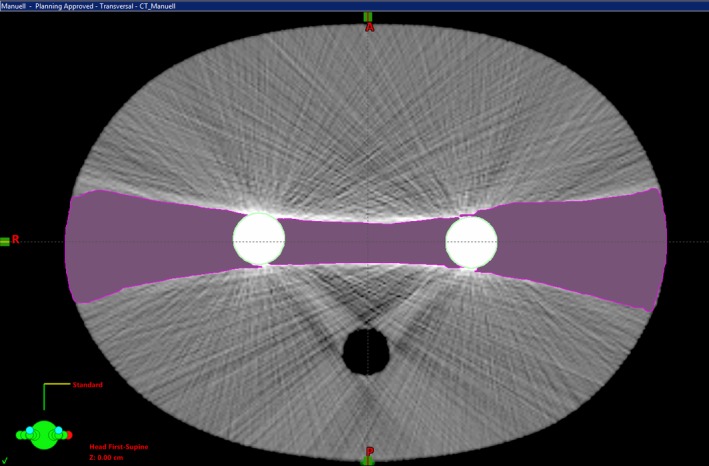
Manually corrected artifacts.

A manual correction of the artifacts is performed using the contouring module of the treatment planning system (TPS) Eclipse™ Version 13.0.26 (Varian Medical Systems, Palo Alto, CA, USA). In the relevant correction region, a volume of interest (VOI) is defined, which encloses the artifacts. Within this VOI, an image threshold filtering is applied in a range of −1000 to 0 HU in order to segment the artifacts. In this segmented area, the HUs are replaced by a constant value of 119 HU since this is the calculated average value of the phantom material in the case of no artifact disturbance (Fig. 2).

## 3 TREATMENT PLANNING

**Figure 3:**
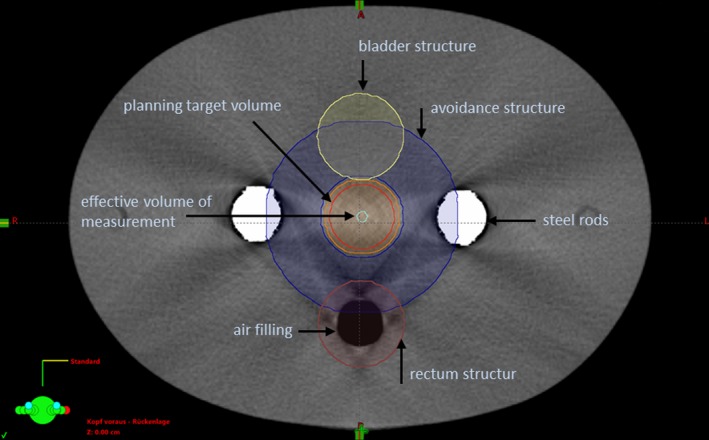
Transversal view ALIR reconstructed phantom CT with metal rods and contoured structures.

Using the TPS Eclipse™, a planning target volume (PTV) approximating a prostate gland was defined with a diameter of 3.0 cm and a length of 3.0 cm. An avoidance structure was introduced which encloses the PTV. By choosing appropriate dose objectives in the dose planning process, it supports the achievement of a steep dose gradient. The effective measuring volume of the ionization chamber was contoured with a diameter of 0.5 cm and a length of 2.0 cm. A bladder volume with a diameter of 4.0 cm and a length of 3.0 cm was created ventrally to this PTV at a distance of 2 mm. Dorsal to the bladder, a rectum structure was generated, which had a diameter of 4.0 cm and a length of 5.0 cm. The distance to the PTV is 1.5 cm. The setup is illustrated in Fig. 3.

The main dose planning objective is to achieve a PTV dose coverage according to the ICRU Report 50 with 107%/95% of the prescribed dose within the PTV.^1^ The VMAT is chosen, as it is the routine method for this entity. In comparison to intensity‐modulated radiotherapy, its ability in sparing organs at risk is more efficient, the account of monitor units is lower, and the target dose distribution is more homogeneous.^18^, ^19^, ^20^

**Figure 4:**
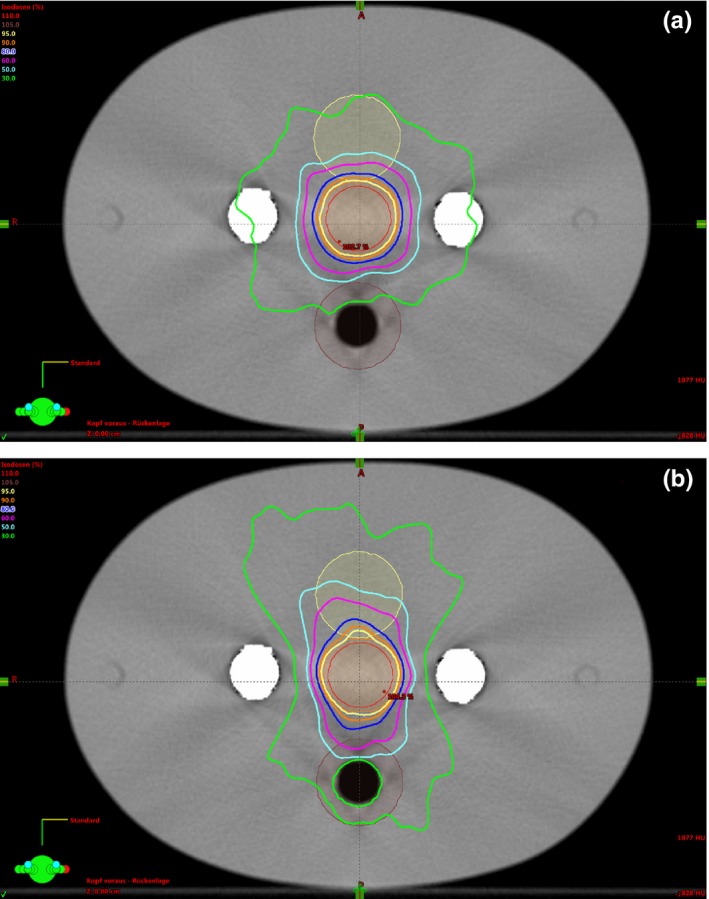
(a) Dose distribution of a VMAT plan with full arc rotation without avoidance sectors. (b) Dose distribution of a VMAT plan with avoidance sectors.

VMAT plans were created in such a way that the 95% isodose covers the PTV. The absorbing effect of the steel rods is taken into account by two different approaches. In the first approach, the issue with the dense material of the rods was left entirely to the optimization tool of the planning software. Here, the linear accelerator rotates 360° with an activated beam around the phantom. In the second approach, avoidance sectors were selected for the rotational angles at which the PTV was covered by the absorbing steel rods [250° to 290° and 70° to 110°, see Figs. 3(a) and 3(b)]. Within these sectors, the object was not irradiated (Figs. 4 a & 4b).

The dose optimization was performed with the VMAT optimization module of Eclipse™, whereby the optimization process was performed in two steps. First, the intermediate dose calculation was performed using the pencil beam algorithm (PBC 10028) 21 and second, the final dose calculation was performed using the anisotropic analytical algorithm (AAA 10.0.28).^22^ The used dose optimization objectives are listed in Table 1. The upper and lower objectives describe the percentage, which should not be exceeded (upper objective) or not fall below (lower objective). A preset priority value weights the importance of the volume in question during the optimization process.

**Table 1:** Dose optimization objectives

Structure	Objective	Volume [%]	Dose [Gy]	Priority
PTV	Upper	0	51.5	350
	Upper	2	51.5	400
	Lower	98	49	400
	Lower	100	49	450
	Mean		50	450
PTV+Margin	Upper	0	51.5	350
	Upper	2	51.5	400
	Lower	98	49	400
	Lower	100	49	450
	Mean		50	450
Avoidance	Upper	0	48	600

### 3.A Dose homogeneity index *HI*

For determination of the homogeneity index, we used the following definition

$$HI=\frac{D_{2\%}-D_{98\%}}{D_{50\%}}$$

where the parameter *D*_x%_ represents the absorbed dose received by x% of the PTV. An *HI* of close to zero indicates that the absorbed dose distribution is almost homogenous.^23^, ^24^

### 3.B Doses in bladder and rectum

For the planning scenario of two rotations without avoidance sectors, applying artifact correction reduces the doses at bladder and rectum. For the bladder and rectum, the mean doses are significantly reduced for all reduction types compared to no correction. For the bladder, the best reduction in mean dose is achieved with LI (−2.7%) and ALIR (−2.3%). Manual correction leads to a reduction of −1.7%. For the rectum, the most reduction in the mean dose is achieved by ALIR (−3.1%) and LI (−2.7%). Manual correction leads to a reduction of −1.8%. (Table 2a & b)

**Table 2:** Mean doses for (a) bladder and rectum without avoidance sectors, (b) bladder and rectum with avoidance sectors

Correction method	Bladder mean dose [%]	Rectum mean dose [%]
(a) Two rotations without avoidance sectors		
No correction	47.1	22.8
ALIR	44.8 (−2.3)	19.7 (−3.1)
LI	44.4 (−2.7)	20.1 (−2.7)
Manual	45.4 (−1.7)	21.0 (−1.8)
(b) Two rotations with avoidance sectors		
No correction	54.8	27.8
ALIR	55.2 (−0.4)	25.9 (1.9)
LI	54.3 (0.5)	27.0 (0.8)
Manual	53.3 (1.5)	26.5 (1.3)

For the planning scenario of two rotations with avoidance sectors, we observe a dose shift after applying artifact correction as well. However, the manifestation is not as distinctive as in the scenario without avoidance sectors. The bladder mean dose shifts for ALIR by −0.4%, for LI by −0.5%, and for manual by −1.5% after correction. The mean doses at the rectum are reduced for ALIR by −1.9%, for LI by −0.8%, and for manual by −1.3%.

## 4 RESULTS

**Figure 5:**
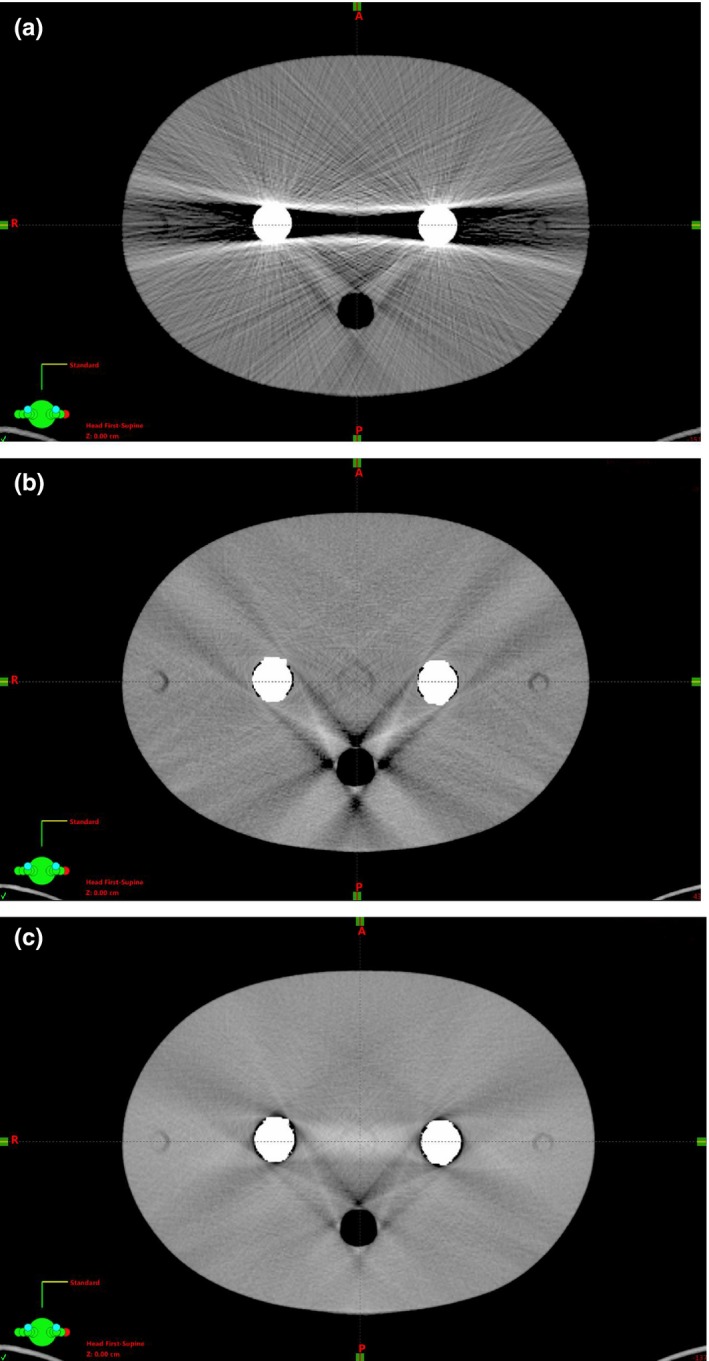
(a) No correction. (b) LI correction. (c) ALIR correction.

Figures 5(a)–5(c) show CT images of representative slices with artifacts as well as after application of LI or ALIR reconstruction. Compared to the LI reconstruction, the ALIR reconstructed image shows fewer streak artifacts.

The isocentric dose measurement shows that artifact‐afflicted image data lead to a significant dose deviation between TPS calculated and measured doses. For the double arc rotations without avoidance sectors and not corrected image data, the maximum error is 8.4%. Image correction with ALIR reduces this error to 2.7%, a correction with linear interpolation to 3.2%. Manual editing of the artifacts reduces the deviation to 4.3%. For double arc rotation with avoidance sectors, the maximum error is 6.4%, reduced to 3.4% by ALIR, 3.5% by LI, and 4.1% by manual correction. The results are listed in Table 3a and b.

**Table 3:** Doses at the isocenter measured with an ionization chamber, calculated mean value, and resulting deviation for (a) two rotations without avoidance sectors, (b) two rotations with avoidance sectors

Correction method	Measured [Gy]%	Mean calculated [Gy]	Δ Meas/cal [%]
(a) Two rotations without avoidance sectors			
No correction	2054	1982	8.4
ALIR	2033	1971	2.7
LI	2030	1967	3.2
Manual	2036	1974	4.3
(b) Two rotations with avoidance sectors			
No correction	2088	2004	6.4
ALIR	2071	1984	3.4
LI	2065	1991	3.5
Manual	2052	1980	4.1

Table 4a and b shows the results for the dose homogeneity index *HI* for each reconstruction method. For both scenarios, the uncorrected FBP reconstruction results in the highest *HI* value. The *HI* for ALIR, LI, and the manual correction are significantly lower. However, all values are close together.

**Table 4:** Homogeneity indices for (a) two rotations without avoidance sectors, (b) two rotations with avoidance sectors

Correction method	D2%	D50%	D98%	*HI*
(a) Two rotations without avoidance sectors				
No correction	2054	1982	1921	0067
ALIR	2033	1971	1924	0055
LI	2030	1967	1923	0054
Manual	2036	1974	1927	0055
(b) Two rotations with avoidance sectors				
No correction	2088	2004	1936	0076
ALIR	2071	1984	1937	0068
LI	2065	1991	1935	0065
Manual	2052	1980	1925	0064

## 5 DISCUSSION

In order to provoke severe streaking artifacts as well as reaching total starvation of the x ray, the diameter of the steel inserts was chosen to be nearly two times the size of a realistic sheath. Nevertheless, it was not possible to reach error values of over 20% as reported from Spadea et al.^11^ However, with an error range in dose calculations from 6% to 8% for uncorrected image data, our results are close to an error range of approx. 5% as reported by Baer et al.^9^

The scenarios we have calculated are normalized in the way that the minimum dose enclosing the PTV corresponds to 95% of the prescribed dose. The corresponding mean and maximum values vary in a narrow range from case to case. The maximum values are in a range of 104.1–107.8%. A mathematical underestimation of the dose leads to a corresponding shift of the real dosage values to higher values. For example, in the worst case, that is, uncorrected CT, calculated for two rotations without avoidance sectors, the dose maximum can be shifted from 105.8% to 114.2%, which consequently means overdosing.

Furthermore, it can be stated that for dose calculations based on ALIR reconstructed images, the best accordance between measured and calculated doses values were reached. One reason for this is the fact that ALIR is able to correct streaking artifacts more efficiently than the LI algorithm, due to its elaborated replacement of corrupted projection values and integrated conditions (see Figs. 5(b) and 5(c) and reference 15).

The homogeneity indices of the investigated scenarios are between values of 0.054 and 0.076. Since the values are very close to zero, even for the images disturbed by artifacts, it indicates a relatively homogeneous dose distribution in all our scenarios. This results from the very good dose coverages of the PTV within a range of 95% of the dose at the minimum and 107% of the dose at the maximum. Due to the steep dose gradient, the values for D_2%_, D_50%_, and D_98%_ are very close together, resulting in very good dose homogeneity. Certainly, these are simplified phantom considerations; real patient treatment plans might differ. However, some interesting tendencies can be observed. All results show that the use of avoidance sectors leads to a reduction in dose homogeneity regardless of the reconstruction procedure. For instance, for the ALIR reconstruction and the case of double rotation with no avoidance sectors, the *HI* is 0.055, but it increases to 0.068 when introducing the avoidance sectors. Furthermore, for the planning with avoidance sectors, the mean doses in bladder and rectum are in general higher compared to the planning scenario without avoidance sectors. This can be understood by illustrating the isodose profiles of both planning scenarios. When planning with avoidance sectors, the isodoses are more cranio‐caudal, while in the planning without avoidance sectors, the isodoses also run laterally. Due to this fact, the doses in the bladder and rectum are higher.

For the artifact correction, a comparison of the two tested scenarios shows a clear shift in values from −2.7% for LI and −2.3% for ALIR for the scenario without avoidance sectors and to 0.5% for LI and −0.4% for ALIR for the scenario with avoidance sectors. These differences can be explained by the location of the bladder contour within the phantom. The bladder is located in an area barely affected by artifacts. This means that the HU values in this area are only slightly changed by ALIR and LI corrections. Therefore, the calculation of the dose results in similar values. In the case of manual correction, a constant HU value is calculated for the substitution of the artifacts. This value differs from the locally available values in a range of about 150–250 HUs. This explains why, in the case of manual correction, a slightly higher dose difference of 1.5% can be seen. The mean doses for the rectum show the same tendencies as described above for the bladder doses. After performing artifact correction, the changes to the uncorrected case for planning with avoidance sectors are lower than for the scenario without avoidance sectors. It is noticeable that the value for LI approaches almost to zero (−0.8%), but not the value for ALIR (−1.9%), as it is the case for the calculation of the bladder. This is because of the severe streaking artifacts caused by the rectum structure, which can be reduced more efficiently by ALIR compared to LI.

When applying arc rotations without avoidance sectors, dose backscatter and attenuation effects due to high‐density TEP material should be considered. For example, a static photon field irradiation of a region shielded by high‐density object leads to an increased dose in front of the material due to backscatter radiation and a decreased dose behind the objects due to attenuation effects.^25^, ^26^ In our scenarios, static photon fields are not used. To what extent the above‐mentioned effects play a role in VMAT techniques with full rotations will be the subject of further investigations. It is quite possible that due to the full rotation and the dynamic MLC movement, the effects are attenuated.

## 6 CONCLUSION

The influence of metal artifact correction on dose calculation in radiation therapy is investigated. It is shown that image reconstruction and correction of artifacts with ALIR lead to a lower error in calculation compared to LI or a manual correction.

An omission of artifact correction leads to an error in the calculation of the isocenter dose and thus leads to a miscalculated dose in the PTV. Furthermore, the doses in cranial and caudal tissue are calculated incorrectly. In both regions, the dose is represented as too high by the planning system, which results in an overdosing in the PTV. Consequently, this means that in order to achieve a higher therapeutic effect in the PTV, the surrounding normal tissue could be dosed higher applying avoidance sectors in order to avoid irradiation of the steel inserts results in a more accurate dose calculation in normal tissue. However, a large error in the isocenter dose is induced. This error is more efficiently adjusted by applying artifact corrections than using rotations without avoidance sectors. Most effective was the application of the ALIR algorithm. If there is no possibility to use automated correction algorithms, artifacts can be overwritten manually with constant HU values. We do not see any relevant influence of avoidance sectors on the dose calculation for manual correction, where the error in the calculation of the isocenter dose differs ca. 4% in both cases.

Furthermore, negligence of artifacts results in a deterioration of the homogeneity in the proposed model. For all investigated methods, the *HI* value is lowest if avoidance sectors are omitted.

## CONFLICTS OF INTEREST

All authors declare that there are no conflicts of interest.
